# Adjuvants influence the immune cell populations present at the injection site granuloma induced by whole-cell inactivated paratuberculosis vaccines in sheep

**DOI:** 10.3389/fvets.2024.1284902

**Published:** 2024-01-30

**Authors:** Miguel Criado, Luis E. Reyes, Juan F. García Marín, Daniel Gutiérrez-Expósito, David Zapico, José Espinosa, Valentín Pérez

**Affiliations:** ^1^Departamento de Sanidad Animal, Facultad de Veterinaria, Universidad de León, León, Spain; ^2^Instituto de Ganadería de Montaña (CSIC-ULE), Finca Marzanas-Grulleros, León, Spain

**Keywords:** adjuvants, paratuberculosis, vaccine injection-site, granuloma, Gudair, Silirum

## Abstract

Vaccination is the most effective tool for paratuberculosis control. Currently, available vaccines prevent the progression of clinical disease in most animals but do not fully protect them against infection and induce the formation of an injection site granuloma. The precise mechanisms that operate in response to vaccination and granuloma development, as well as the effect that adjuvants could trigger, have not been fully investigated. Therefore, this study aimed to investigate the injection site granulomas induced by two inactivated paratuberculosis vaccines, which differ in the adjuvant employed. Two groups of 45-day-old lambs were immunized with two commercially available vaccines—one (*n* = 4) with Gudair^®^ and the other (*n* = 4) with Silirum^®^. A third group (*n* = 4) was not vaccinated and served as control. The peripheral humoral response was assessed throughout the study by a commercial anti-*Mycobacterium avium* subspecies *paratuberculosis* (*Map*) antibody indirect ELISA, and the cellular immune response was assessed similarly by the IFN-γ release and comparative intradermal tests. The injection site granulomas were measured during the experiment and sampled at 75 days post-vaccination (dpv) when the animals were euthanized. The tissue damage, antigen and adjuvant distribution, and the presence and amount of immune cells were then determined and assessed by immunohistochemical methods. Antibodies against *Map* antigens; a general macrophage marker (Iba1), M1 (iNOS), and M2 (CD204) macrophages; T (CD3), B (CD20), and γδ T lymphocytes, proteins MHC-II and NRAMP1, and cytokines IL-4, IL-10, TNF, and IFN-γ were employed. Silirum^®^ elicited a stronger peripheral cellular immune response than Gudair^®^, while the latter induced larger granulomas and more tissue damage at the site of injection. Additionally, adjuvant and *Map* antigen distribution throughout the granulomatous inflammatory infiltrate, as well as the NRAMP1 cell expression, which is linked to antigen phagocytosis, were highly irregular. In Silirum^®^ induced granulomas, a higher number of MHC-II and TNF-expressing cells and a lower number of M2 macrophages suggested an improved antigen presentation, which could be due to the better antigen distribution and reduced tissue damage induced by this vaccine.

## 1 Introduction

Paratuberculosis (PTB) is a chronic infectious disease caused by *Mycobacterium avium* subsp. *paratuberculosis* (*Map*) characterized by granulomatous enteritis. It affects ruminants worldwide and is linked to significant economic losses (1). The long incubation period and subclinical character of this disease, along with the lack of highly sensitive diagnostic tests, make the detection of infected animals extremely difficult (2). Control measures based on testing and culling have proven to be expensive and not entirely successful (3). Therefore, vaccination is regarded as the most efficient control procedure in terms of cost-benefit (4).

At this moment, only inactivated vaccines for PTB control are commercially available. Gudair^®^ and Silirum^®^, produced by Zendal (Porriño, Spain), are approved for their use in small ruminants and cattle, respectively. Mycopar^®^, manufactured by Boehringer Ingelheim Vetmedica Inc., has been recently discontinued (2019) in the United States (5). These vaccines are subcutaneously administered and present several disadvantages, as they all induce severe granulomatous chronic inflammation at the injection site (6–8) and interfere with the tuberculosis diagnostic tests (9, 10). Even though they reduce clinical signs, bacterial shedding, and economic loss in the flock or herd, they do not prevent *Map* infection and do not provide full protection for all vaccinated animals (11, 12). Granuloma formation has been mainly attributed to the mineral oils employed as adjuvants in these vaccines, whose objective is to produce an emulsion with the bacteria and be able to induce a strong and effective immune response (13). However, due to their limited biodegradability and low biocompatibility, they can persist for long periods of time, triggering a chronic immune response with macrophage recruiting. As a side effect, a granuloma appears in the injection site, which impacts animal welfare, carcass quality, and even biomedical research (7, 14). Yet, these granulomas are poorly studied, not only in PTB vaccines but in all oil-adjuvanted vaccines.

The main mechanism behind oil-based adjuvants is thought to be the slow release of the antigen at the injection site (depot effect) (15, 16). Successful induction of immunologic memory starts there, with the local activation of the innate immune system. Following injection, activated resident immune cells produce pro-inflammatory cytokines and chemokines, which induce the recruitment of other immune cells, including antigen-presenting cells (APC). After maturation, APC expressing higher levels of the major histocompatibility complex class II (MHC-II) and other co-stimulatory molecules migrate to the draining lymph nodes, where they activate potent antibody-secreting B cell and/or T cell responses. Adjuvants facilitate most steps of this process, but the particular mechanisms that determine the effect of the different adjuvants are poorly understood (16, 17).

Therefore, the characterization of the cell populations present at the vaccine inoculation site is crucial to understanding the development of the granulomas at the injection site (18) and the establishment of the immune response after vaccination (19). Macrophages are the main component of any granulomatous lesion (20), and their role as APC is essential in the development of the immune response (21, 22). Macrophages present extraordinary plasticity and have been traditionally classified into M1 (classically activated) and M2 (alternatively activated) types, in an analogy to the CD4+ T cell's Th1 and Th2 cytokine responses (23–25). M1 cells are mainly activated by interferon-γ (IFN-γ), while M2 cells are activated by interleukins 4 and 10 (IL-4 and IL-10) (23). They can be distinguished by the expression of different markers, which can be functional, such as inducible nitric oxide synthase (iNOS) and tumor necrosis factor-α (TNF) for M1 macrophages or membrane proteins such as CD204 for M2 macrophages. However, this classification, established *in vitro*, is rather simplistic and outdated, and a broader range of differentiation and functions is being identified (26, 27). Macrophages also variably express major histocompatibility complex (MHC) class II, a molecule essential in initiating immune responses. MHC class II is also expressed in dendritic cells, monocytes, and B-lymphocytes, which are other main APCs in the skin's immune system (28, 29). Another essential protein expressed by macrophages is the natural resistance-associated macrophage protein 1 (NRAMP1), an integral protein of the lysosome membrane that is targeted by the antigen-containing phagosome (30) and has been related to the resistance against mycobacterial infections (31). On the other hand, lymphocytes play a secondary role in the development of granulomas and are influenced by the cytokine environment generated by activated macrophages. Further, it is also assumed that Th1 and Th2 T cells impact macrophage polarization toward M1 and M2 profiles, respectively (32).

In the present study, we employed two of the currently approved vaccines against PTB, which were formulated with the same antigen (inactivated *Map* strain 316F) but different adjuvants—Gudair^®^ employs a mineral oil adjuvant and Silirum^®^ a water-in-oil-in-water (W/O/W) adjuvant, constituted by organic and more refined biocompatible mineral oils. The histopathological evaluation of the pathological features of the vaccination nodules, mainly the inflammatory response, necrosis, and tissue damage induced by these vaccines at the inoculation point, is of great interest. Given that these oil-based vaccines induce severe tissue damage, damage-associated molecular patterns (DAMPs) are also of equal interest. These molecules can be found in normal cells and are released under tissue damage or stress; therefore, they could play an important role in adjuvanticity (33). Additionally, these adjuvants not only act as immunomodulators but also as delivery systems, influencing the distribution of the antigen (34).

The objective of this study is to evaluate and compare the histopathological features, antigen distribution, and immune cells present in the granulomas induced by two different PTB vaccines at 75 days post-vaccination (dpv) at the subcutaneous inoculation site in the ovine species. The study also focuses on the effect of these granulomas on the specific humoral and cellular peripheral immune responses associated with vaccination at this time point.

## 2 Materials and methods

### 2.1 Animals and experimental design

A total of twelve 1.5-month-old lambs of the Churra breed were used in this study. The animals were randomly selected and acquired from a commercial herd in which no clinical cases of PTB or any other relevant diseases had been reported in the last 5 years and kept in the experimental facilities of the Instituto de Ganadería de Montaña CSIC-ULE (Grulleros, Spain) throughout the experiment. The lambs and their dams were tested as negative for PTB antibody ELISA using the commercial kit ID Screen^®^ Paratuberculosis Indirect (IDvet, Gabrels, France) and for the interferon-γ (IFN-γ release assay (IGRA), the BOVIGAM™ TB Kit (Thermo Fisher Scientific, Waltham, USA) (35). After 2 weeks of an adaptation period, they were divided into three groups, composed of four animals each. All the animals followed a diet based on grass hay *ad libitum*, supplemented with a rationed conventional compound feed, and had free access to water.

Animal handling and sample collection were carried out in accordance with European Union legislation (Law 6/2013) concerning animals, their exploitation, transportation, experimentation, and sacrifice, Royal Decree 118/2021 for the protection of animals employed in research and teaching, Directive 2010/63/UE, related to the protection of animals used for scientific goals. All the procedures were approved by the corresponding animal welfare body (OEBA) and the Consejería de Agricultura y Ganadería de la Junta de Castilla y León (authorization code ULE-02–2021). All animals used in this study were handled in strict accordance with good clinical practices, and all efforts were made to minimize suffering.

### 2.2 Vaccines and inoculation

Animals belonging to the first group (*n* = 4) were inoculated with Gudair^®^ (Zendal, Porriño, Spain), which uses the mineral oil Marcol^TM^ 52 (ExxonMobil, Irving, TX, USA) in a stable double suspension, and the heat-inactivated 316F *Map* strain—an attenuated strain—at a concentration of 2.5 mg/ml. The second group (*n* = 4) was vaccinated with Silirum^®^ (Zendal, Porriño, Spain), which uses the same antigen and amount, but a water in oil in water (W/O/W) adjuvant from the Montanide^TM^ ISA range (Seppic S.A., Paris, France), formed by a mixture of mineral and metabolizable oil. The inoculation was performed at 45 days of age, with a single dose of 1 ml of each respective vaccine, subcutaneously in the right caudolateral neck region. Finally, the remaining animals constituted the non-vaccinated control group (*n* = 4).

### 2.3 Clinical examination and sampling

Blood sampling was carried out at 0 dpv, 15 dpv, 30 dpv, and 75 dpv. Every time, two jugular blood samples were taken in Vacutainer tubes (Becton Dickinson, UK), one in a 5 ml tube without additives to obtain serum and another in a 10 ml tube with heparin.

At 1 dpv, 2 dpv, 7 dpv, 15 dpv, 21 dpv, 30 dpv, and 75 dpv, the animals underwent clinical examination, and the injection site nodule was measured using a caliper (length and width) and evaluated, checking the consistency, signs of pain, surface texture and the presence of adherences or ulceration.

At 75 dpv, the lambs were humanely euthanized by deep sedation with xylazine (Xilagesic^®^, Calier, Barcelona, Spain) and subsequent intravenous injection of embutramide, mebezonium iodide, and tetracaine hydrochloride (T61^®^, MSD Animal Health), followed by exsanguination. A complete necropsy was performed, and the injection site nodule was taken (except in the animals from the control group, where it was absent) from all the animals for histopathological and immunohistochemical examination. The tissue samples were fixed in 10% neutral buffered formalin and dehydrated through a graded alcohol series before being embedded in paraffin wax.

### 2.4 Determination of the humoral immune response by indirect ELISA

The ID Screen^®^ Paratuberculosis Indirect (IDvet, Gabrels, France) ELISA test was employed to measure the specific antibody levels in the sera obtained from the blood samples without anticoagulant. The test was performed following manufacturer instructions, and for interpretation, the optical density was measured spectrophotometrically at a wavelength of 450 nm (OD_450_), and the results were expressed as a ratio between the mean OD_450_ of each sample sera duplicates and the mean OD_450_ of the positive control sera duplicates in each plate.

### 2.5 Interferon-γ release assay

For each animal, within 3 h after blood collection, two 1.5 ml aliquots of the heparinized blood samples were incubated in 24-well sterile plates with either 100 μl of sterile phosphate-buffered saline (PBS) or an avian purified protein derivative (PPDa) antigen (CZ Veterinaria, Porriño, Spain), at a final concentration of 20 μg/ml. After incubation (20 h at 37°C), plates were centrifuged at 750 g for 15 min, and plasma was collected and stored at−20°C (36– 38). Then, the assay for IFN-γ determination BOVIGAM^®^ TB Kit (Thermo Fisher Scientific, Waltham, USA) was carried out following the manufacturer instructions, interpreted as previously described (37), and results expressed as a quotient between the mean OD of the PPDa-stimulated plasma and the mean OD of the same plasma incubated with PBS.

### 2.6 Comparative intradermal test

The comparative intradermal test was carried out at 75 dpv. After measuring the caudal folds with a cutimeter, 0.1 ml of PPDa (CZ Veterinaria, Porriño, Spain) and 0.1 ml of bovine purified protein derivative (PPDb) (CZ Veterinaria, Porriño, Spain), obtained from 2500 UI of *Mycobacterium avium* strain D4 and *Mycobacterium bovis* strain AN-5 respectively were injected in the left and right caudal folds. After 72 h, the folds were measured again, and the results were expressed as the difference between this measurement and the initial thickness in mm.

### 2.7 Histopathology

For the histopathological evaluation of the injection site nodule, 3 μm thick sections were stained with hematoxylin and eosin (HE) and with the Ziehl–Neelsen method for acid-fast bacilli (AFB) detection and examined under an optical microscope by two veterinary pathologists (MC and VP), blinded to the animal ID.

### 2.8 Immunohistochemistry

Immunohistochemical studies of each vaccination nodule were performed on 3 μm-thick tissue sections, placed onto poly-L-lysine-coated slides (SuperFrost Plus Adhesion slides -Thermo Fisher Scientific, Waltham, USA-). After deparaffination and hydration, the sections were washed two times using wash buffer (Agilent Technologies, Santa Clara, USA) for 5 min. Then, endogenous peroxidase was blocked by immersion of the sections into a 3 % H_2_O_2_ in methanol for 30 min in darkness at room temperature, washed again, and antigen retrieval was performed using heat-based or enzymatic methods, as stated in Table 1. After washing two times, the sections were incubated with the primary antibodies (listed in Table 1), diluted in antibody diluent (Agilent Technologies, Santa Clara, USA), overnight at 4°C in a humidified chamber. After washing, sections were incubated for 40 min at room temperature with the appropriate monoclonal or polyclonal antibody and horseradish peroxidase-labeled polymer (Agilent Technologies, Santa Clara, USA). Antibody localization was then determined using 3, 3-diaminobenzidine (Agilent Technologies, Santa Clara, USA) as a chromogenic substrate for peroxidase. Sections were counterstained with Mayer's hematoxylin for 10 s. Appropriate species- and isotype-matched immunoglobulins were used as negative controls. Depending on the antibody employed, lymph nodes from healthy sheep or ileocecal valves from *Map-*infected sheep were used as positive controls.

**Table 1:** Primary antibodies, unmasking technique, and dilution used for immunohistochemistry.

**Target**	**Clone/Reference**	**Epitope demasking**	**Dilution**	**Supplier**
*Mycobacterium avium* subspecies *paratuberculosis* strain 2E	B312	Trypsin 0.1 %, 15 min	1:5000	DakoCytomation, (Glostrup, Denmark)
Bovine WC1 (workshop cluster 1)	CC15	None	1:200	Bio-Rad Laboratories Inc. (Hercules, CA, USA)
Human CD20	RB9013P	None	1:200	ThermoFisher Scientific, Waltham, USA
Human CD3	A0452	HIER, pH 6.0	1:300	Agilent Technologies (Santa Clara, CA, USA)
Human CD204	SRA-E5	HIER, pH 6.0	1:400	TransGenic (Kumamoto, Japan)
Ovine MHC-II (major histocompatibility complex class II)	VPM36	HIER, pH 6.0	1:300	Bio-Rad Laboratories Inc. (Hercules, CA, USA)
Rat Iba1 (ionized calcium-binding molecule 1)	019–19741	HIER, pH 6.0	1:2000	Wako (Japan)
Bovine IL-4 (interleukin 4)	CC313	HIER, pH 6.0	1:300	Bio-Rad Laboratories Inc. (Hercules, CA, USA)
Bovine IL-10 (interleukin 10)	CC318	HIER, pH 9.0	1:100	Bio-Rad Laboratories Inc. (Hercules, CA, USA)
Human NRAMP1 (natural resistance-associated macrophage protein 1)	SC20113	HIER, pH 6.0	1:50	Santa Cruz Biotechnology, Inc. (CA, USA)
Bovine TNF-α (tumor necrosis factor α)	CC327	HIER, pH 6.0	1:250	Bio-Rad Laboratories Inc. (Hercules, CA, USA)
Murine iNOS (inducible nitric oxide synthase)	NB300–605	Trypsin 0.1 %, 15 min	1:200	Novus Biologicals (Littleton, CO, USA)
Bovine IFN-γ (interferon-γ)	CC330	HIER, pH 9.0	1:100	Bio-Rad Laboratories Inc. (Hercules, CA, USA)

Heat-induced epitope retrieval (HIER) was performed at 95°C for 20 min using PT-link and Dako Target Retrieval Solutions (Agilent Technologies, Santa Clara, CA, USA), and enzyme-induced epitope retrieval was carried out at ambient temperature.

The distribution of the *Map* antigen was assessed qualitatively. Regarding the positively immunolabelled cells for the different markers, their distribution was evaluated in every section, and an adequate approach for cell counting was determined for each marker. Counts were carried out in the areas of granulomatous inflammation, avoiding the connective tissue capsule of the granulomas, as well as the necrotic areas. Counts were carried out in ten randomly selected fields at 400x magnification (high power fields -HPF-). In the case of sections immunostained with workshop cluster (WC) 1 antibody, 30 fields were evaluated per slide, given the scarcity of WC1+ γδ T cells. Additionally, CD20+ cells were distributed throughout the inflammatory tissue in low numbers but formed a dense layer around most granulomas; therefore, ten fields were counted in each of these areas.

Micrographs were taken using the Nikon^®^ Eclipse E600 microscope, coupled with a Nikon^®^ DS-Fi1 digital camera. Images are representative of the granulomas induced by each vaccine unless specified otherwise. Cell counting and image analysis were performed using the Image J processing and analysis software (US National Institutes of Health, Bethesda, Maryland). Evaluation of all immunostaining was performed independently by two pathologists (MC and VP), and discordant results were reviewed with a multiheaded microscope to reach consensus.

### 2.9 Statistical analyses

The results of the different parameters analyzed (antibody ELISA, IGRA and CID tests data, vaccination nodule size, and immunohistochemical counts) according to the different vaccine types inoculated and dpv, are reported as means and standard deviation, calculated using conventional descriptive statistical procedures and represented by bar and line plots. The Shapiro-Wilk test was used to assess data normality. Parametric and non-parametric tests were used depending on the nature of the data.

Specifically, two-way, repeated measure ANOVAs were used for analyzing the effect of the different vaccines on antibody levels, IFN-γ response, and nodule size evolution throughout the study. When a statistically significant interaction between vaccine and time on the studied parameter was detected, the effect of the vaccine was analyzed at each time point. *Post-hoc* analyses, with a Bonferroni adjustment, were used to reveal the pairwise significant differences between groups and time points. A one-way ANOVA was used to analyze the results of the CID test. Significant differences in the caudal fold thickness increase depending on the PPD employed were detected, and Tukey's HSD Test for multiple comparisons was used for *post-hoc* analysis. Additionally, the Pearson correlation test was applied to assess the relationship between antibody levels and IGRA results. For analyzing the immunohistochemistry cell counts, the Mann-Whitney *U* test (in the case of MHC-II, Iba1, iNOS, CD3, IL-4, IFN-γ, and WC1 γδ T cells) and unpaired *t*-test (CD204, NRAMP1, and CD20 cells) were employed to identify the statistically significant differences according to the vaccine employed. *P* < 0.05 were considered statistically significant. The statistical packages rstatix, ggpubr, forcats, stringr, dplyr, purrr, readr, tidyr, tibble, ggplot2, and tidyverse were used in the different statistical tests. All statistical analyses were performed with the R software version 4.1.3 (39).

## 3 Results

### 3.1 Vaccination nodule development, clinical progression, and gross pathology

Apart from the development of a nodule at the injection site, none of the animals showed any clinical signs or systemic adverse reactions to the vaccination during the experiment.

**Figure 1:**
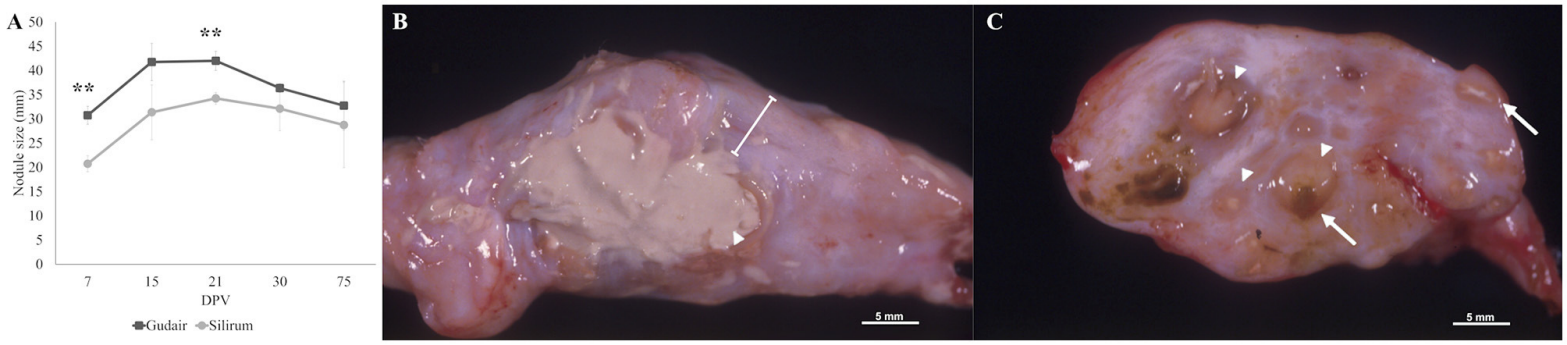
Evolution of the injection-site nodule size and gross morphology by 75 dpv. **(A)** The mean size of the injection-site nodule (in mm) error bars represents standard error. Indicated significant differences are between groups. ^**^p < 0.01. **(B)** Gross morphology of a granuloma induced by Gudair^®^, where a large area of caseous necrosis is surrounded by a thick fibrous capsule (line). **(C)** Gross morphology of a granuloma induced by Silirum^®^, composed of multiple small inflammation foci of variable size (arrowheads) demarcated by conjunctive tissue, some of them show a necrotic center (arrows) and/or adjuvant remnants (yellowish-brown material).

The evolution of the size of the vaccination nodule is represented in Figure 1A. Data are expressed as the mean between the nodule length and width in mm. The nodules were larger in the animals vaccinated with Gudair^®^ throughout the entire study, though this difference was only significant at 7 (*p* < 0.01) and 21 dpv (*p* < 0.01). In the first 48 h, the clinical evolution of the inoculation site was similar for both groups. At 24 h, a mild cutaneous erythema and a local temperature increase were detected. On the second day, a diffuse thickening of variable size and an increase in consistency was noticed in some animals in the injection site. From then on, clinical examinations were carried out weekly for the first 4 weeks and then at 75 dpv. The size, clinical progression, and gross pathology findings of the vaccination nodules are described below.

In the Gudair^®^ vaccinated animals, during the first few days, the nodule thickness increased rapidly, and by 15 dpv, the nodules measured an average of 41.7 mm, and their surface was slightly irregular. In one of the lambs, the thickening affected the underlying muscular tissue and had a wide, poorly defined base. At 21 dpv, most nodules had reached their maximum size, with a mean diameter of 42 mm. At 30 dpv, the nodules were highly irregular in shape, even lobed in one of the lambs, and one of them developed a fistula that released a purulent necrotic material. At 75 dpv, fistulae were present in two animals, and consequently, nodule size varied considerably between individuals (20 mm−43.5 mm). At post-mortem gross examination, nodules had a fibrous aspect and extensive, diffusely distributed areas of caseous necrosis (Figure 1B).

In the animals vaccinated with Silirum^®^, at 7 dpv, the vaccination nodules averaged 20.75 mm. Throughout the entire experiment, they were round, well-demarcated, with a regular surface and firm consistency. At 21 dpv, the nodules reached their maximum size of a mean of 34.25 mm. At 75 dpv, the vaccination nodules were smaller, and one of them showed a fistula. Grossly, small, multiple, numerous necrotic foci of 2 mm−3 mm were present throughout the entire nodule (Figure 1C).

### 3.2 Peripheral immune response

#### 3.2.1 Humoral immune response

**Figure 2:**
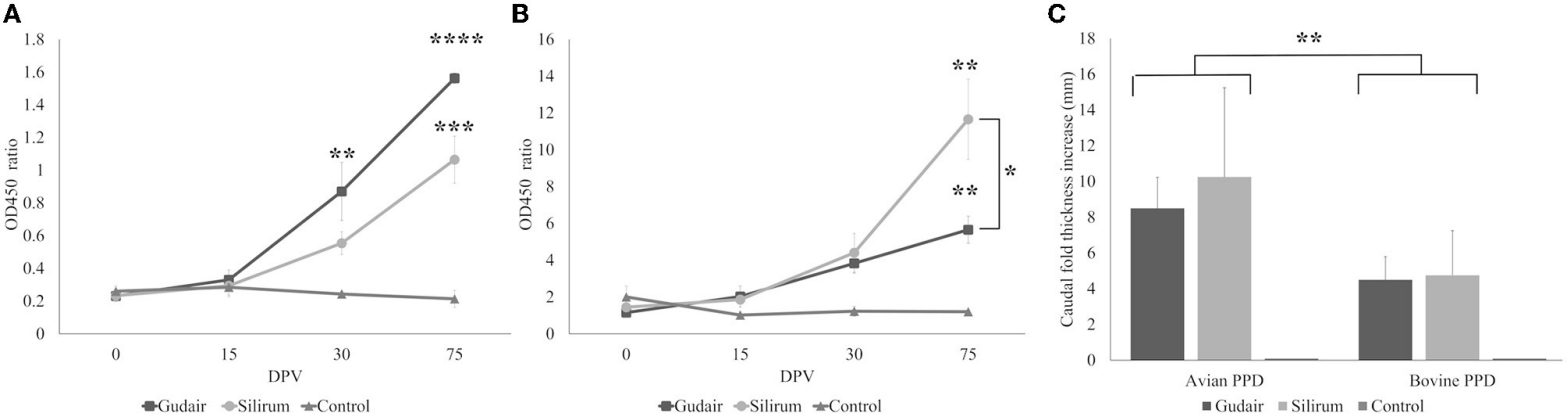
Evolution of the peripheral immune response. Kinetics of **(A)** antibody production and **(B)** interferon-γ released in response to avian PPD throughout the study. Asterisks indicate significant increases within a group with respect to 0 dpv levels; brackets indicate significant differences between groups. **(C)** Results of the comparative intradermal test (CID), expressed as an increase in caudal thickness after inoculation with either avian or bovine PPDs, brackets represent differences between PPDs. Results are expressed as a mean. Error bars represent standard error. *p < 0.05, **p < 0.01, ***p < 0.001, ****p < 0.0001.

The antibody production against *Map* in the different vaccinated groups throughout the experiment is shown in Figure 2A. Both groups showed a statistically significant (*p* < 0.0001) progressive increase in specific antibody levels over time, whereas, for the control group, they remained constant. Within groups, this increase was significant, with respect to the basal levels (0 dpv) in Gudair^®^ vaccinated animals at 30 dpv animals (*p* < 0.01) and at 75 dpv (*p* < 0.0001), while in Silirum^®^ vaccinated animals, this increase was only significant at 75 dpv (*p* < 0.001). Comparatively, from 15 dpv to the end of the experiment, the group vaccinated with Gudair^®^ showed the highest mean antibody titers, and at 75 dpv, they were significantly higher (*p* < 0.05) than that of Silirum^®^ vaccinated animals.

#### 3.2.2 Cellular immune response

The IGRA test results from the different groups are shown in Figure 2B. IFN-γ levels showed a similar pattern of evolution to that of antibody production (correlation coefficient = 0.66; *p* < 0.00001), with a progressive increase throughout the study in both vaccinated groups. By 75 dpv, this increase was significant (*p* < 0.01), with respect to the basal levels (0 dpv) and significantly higher in Silirum^®^ vaccinated animals than in Gudair^®^ (*p* < 0.05). No significant changes were observed in the control group at any point in the study.

On the other hand, the results of the CID test revealed different responses between vaccinated groups (Figure 2C). After injection with bovine and avian PPDs, the two vaccinated groups showed a significant (*p* < 0.01) increase in the caudal fold thickness with respect to the control group, in which the skin reaction was absent. The caudal fold thickness increase induced by PPDa was significantly higher (*p* < 0.01) than that induced by PPDb in both vaccinated groups. In both cases, the most intense response was observed in Silirum^®^ vaccinated animals, but this difference was not significant (*p* > 0.05).

### 3.3 Study of the injection site granulomas

#### 3.3.1 Histopathological findings and antigen distribution

**Figure 3:**
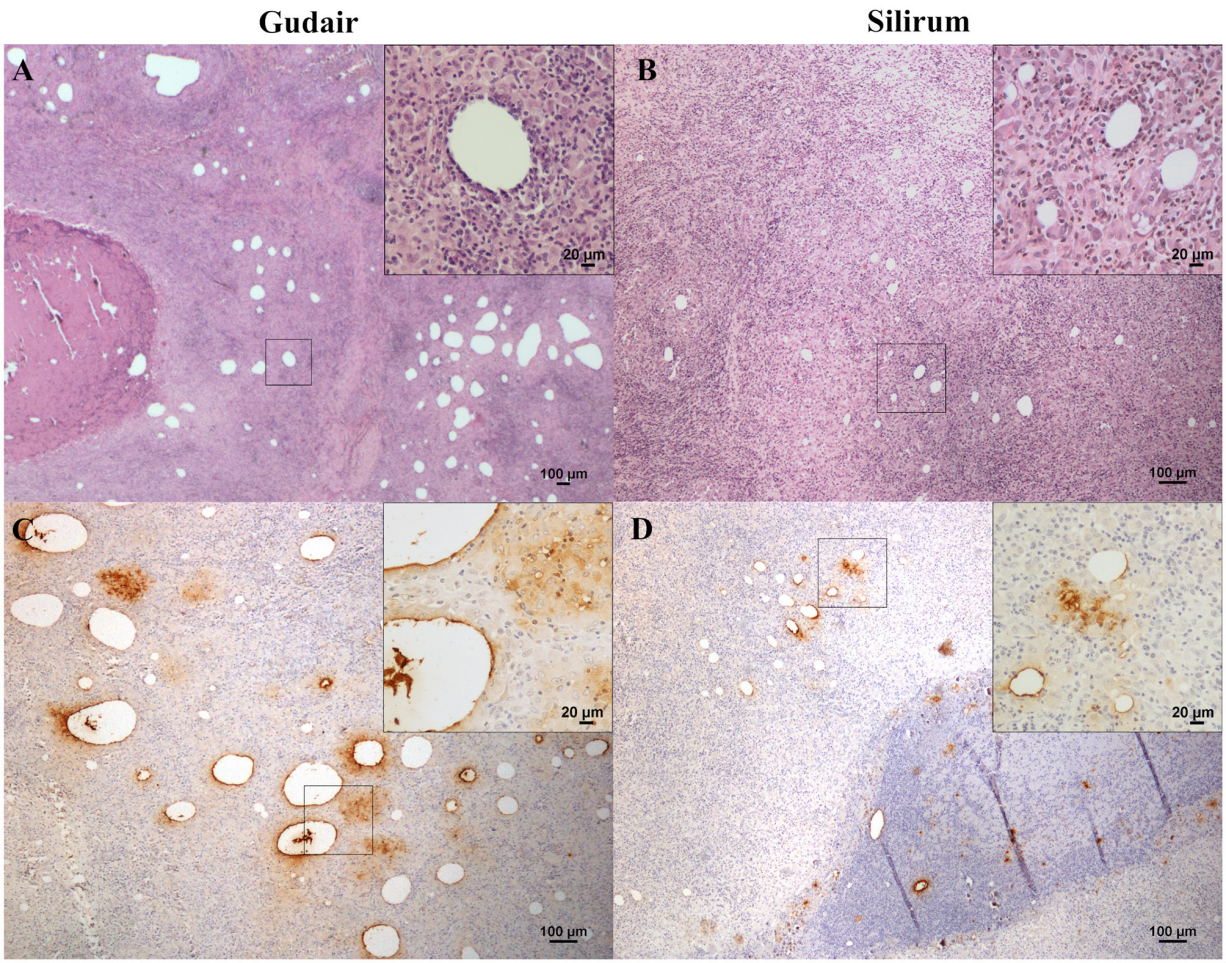
Vaccine-induced granulomas histology and immunohistochemical assessment of antigen distribution. Histology of the granulomatous lesions induced by **(A)** Gudair^®^: droplets of very variable size were distributed throughout the tissue, surrounded by an inflammatory reaction composed of macrophages and lymphocytes (inset). The granulomatous areas are encapsulated by fibrous tissue. In the left corner, a necrotic area induced by the vaccine can be observed. **(B)** Silirum^®^: smaller vaccine droplets of homogeneous size are distributed throughout the tissue and surrounded by granulomatous tissue of similar cellular composition (inset). *Map* antigen immunohistochemistry of the granulomas induced by **(C)** Gudair^®^ shows that large amounts of antigen persist in and around some of the vaccine droplets, in the top right corner of the inset, macrophages with internalized adjuvant -clear amorphous material- and/or antigen -immunolabeled- can be seen and; in **(D)** Silirum^®^ group, smaller droplets and lower quantities of antigen can be observed, detail in the inset. Figures are representative of the granulomas induced by each vaccine.

In the Gudair^®^ vaccinated animals, at the vaccine inoculation point, a severe granulomatous inflammation of variable extension, but well demarcated, was observed expanding the subcutaneous tissue. It was composed of abundant macrophages, and Langhans giant cells admixed with some lymphocytes and, to a lesser extent, neutrophils that were seen mainly surrounding several caseous necrotic foci, in which extensive areas of mineralization were frequent. The quantity of lymphocytes and neutrophils present was inversely related, and neutrophil presence was in direct relation to the extent of necrosis and tissue damage. The presence of fibrous tissue was prominent in all the lesions, and not just in the periphery of the granulomatous inflammation area, such that it was also seen encapsulating smaller granulomas within the inflammatory area. In the animals whose nodules evolved into a fistula, an empty cavity communicated with the exterior through a solution of continuity in the skin, surrounded by intense granulomatous inflammation and fibrous tissue, was seen. Among the inflammatory infiltrate, non-stained lipid droplets were detected. They were larger and more abundant than in the Silirum^®^ group (Figure 3A), and the immunolabeling of *Map* revealed that large amounts of antigen persisted in some of them (Figure 3C), in well-defined deposits and in the necrotic foci, where the biggest antigen deposits were found.

In lambs vaccinated with Silirum^®^, the presence of a granulomatous inflammation, demarcated by connective tissue, was also a typical feature. However, fibrosis and necrosis were less extensive, and the inflammatory cellular component predominated over the tissue damage. Smaller, homogeneous, and dispersed vaccine droplets were less abundant than in the previous group and were surrounded by well-differentiated epithelioid cells, Langhans-type giant cells, and a great number of lymphocytes (Figure 3B). Neutrophils were also present in the necrotic areas found in the center of the granulomas. Areas of mineralization were observed in some necrotic areas. *Map* immunolabeling was moderate in the vaccine droplets (Figure 3D), necrotic areas, and also, frequently, in the cytoplasm of the epithelioid and giant cells located in the periphery of the vaccine droplets, denoting an intense phagocytic activity of these cells.

The amount of bacilli stained with the Ziehl–Neelsen method in the vaccine droplets from both groups was very low with only a faint positive staining in some of them (data not shown).

#### 3.3.2 Immunohistochemical assessment of the immune cells present at the injection site granuloma

##### 3.3.2.1 Distribution of labeled cells

**Figure 4:**
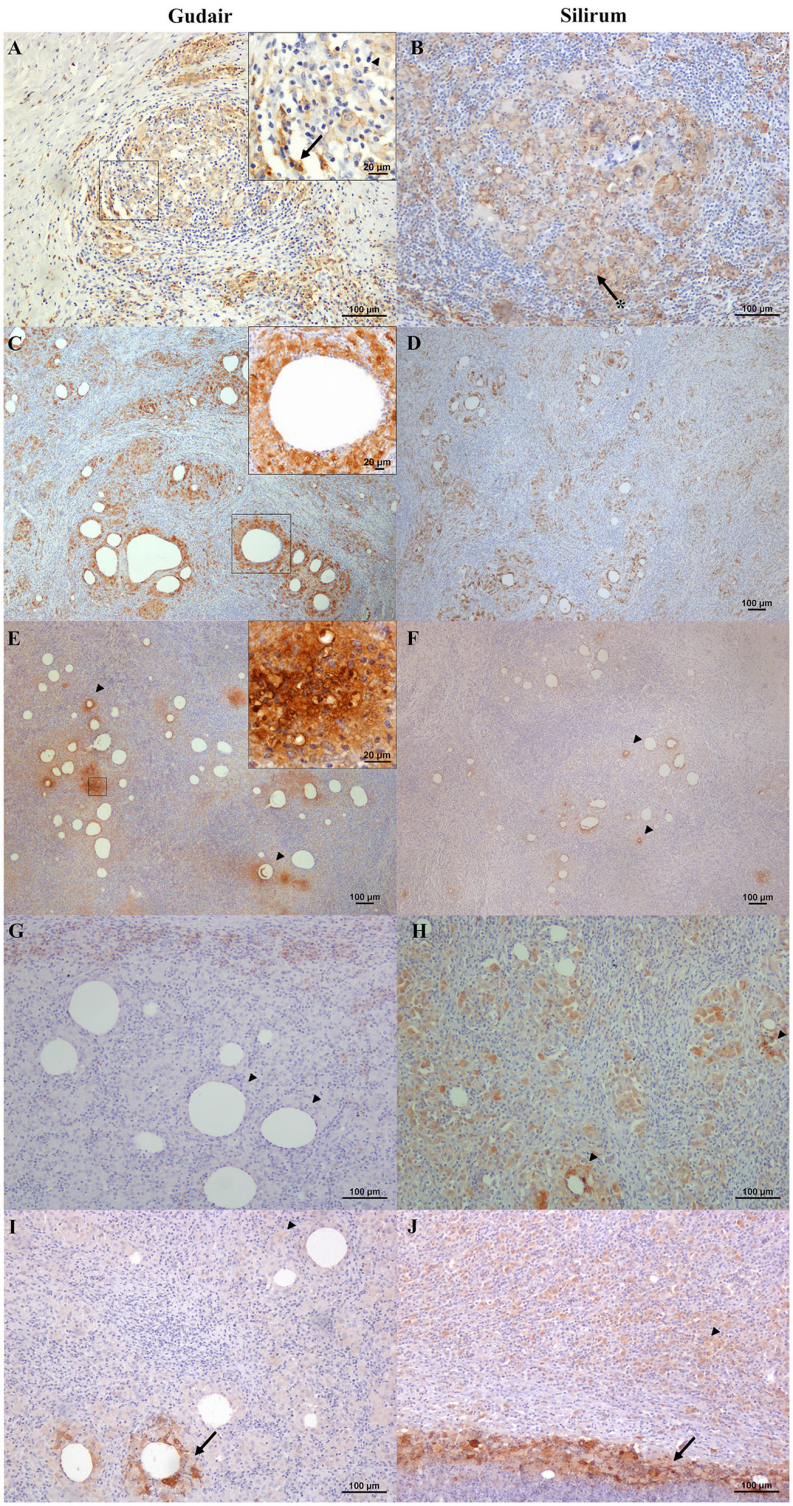
Immunohistochemical expression of macrophage markers in the vaccine-induced granulomas. **(A, C, E, G, I)** Gudair^®^ and **(B, D, F, H, J)** Silirum^®^ vaccines. **(A, B)** Iba1+ cells are present and abundant in the granulomas induced by both vaccines, with a similar distribution. The non-epithelioid macrophages present in the outer layer of the granulomas often show homogeneous strong staining (arrow, inset), whereas, at the center of the granuloma, the epithelioid macrophages (arrowhead, inset) and the Langhans giant cells (asterisk) show a strong membrane staining pattern. **(C, D)** CD204 positively immunolabelled macrophages are present at the first cell layer around vaccine droplets (inset) in the granulomas induced by both vaccines. **(E)** In granulomas induced by Gudair^®^, natural resistance-associated macrophage protein 1 (NRAMP1) is expressed by most epithelioid macrophages, whose staining intensity increases in some cells located at the vicinity of only some of the vaccine drops (arrowheads), **(F)** In those induced by Silirum^®^, NRAMP1 expression shows a similar staining pattern, but the foci of cells showing a high expression are smaller and less abundant (arrowheads). **(G)** Gudair^®^ vaccine. Moderately immunostained TNF+ macrophages can be seen at the periphery of a granuloma formed around vaccine droplets, whereas epithelioid macrophages in the proximity of the droplets were negative (arrowheads), **(H)** Silirum^®^ vaccine. Cells showed strong positive immunolabelling, particularly around some of the vaccine droplets (arrowheads). **(I, J)** iNOS+ macrophages are intensely labeled in the proximity of some of the vaccine droplets and necrotic areas (arrows), whereas most macrophages that form the granulomas show a weaker stain (arrowheads).

Cells labeled with ionized calcium-binding adapter molecule 1 (Iba1), a general macrophage marker, were observed in the center of the granulomas (Figures 4A, B) and were found in direct contact with the necrotic areas or the vaccine droplets. Epithelioid macrophages were intensely labeled with this marker, and most Langhans-type multinucleated giant cells showed a weaker stain. The staining pattern was cytoplasmic, but in the center of the granulomas and around some vaccine droplets, cells also showed intense membranous staining (Figure 4B). The staining pattern in cells immunolabelled with CD204 antibody was cytoplasmatic and more intense in the epithelioid macrophages that surrounded the vaccine droplets and the necrotic center of the granulomas (Figures 4C, D). Multinucleated giant cells were variably labeled. NRAMP1 immunolabelling showed a cytoplasmatic staining pattern and was expressed in epithelioid macrophages with variable intensity, as well as in giant cells. Differences in the staining intensity of NRAMP1+ macrophages between groups could be observed. In Gudair^®^ vaccinated animals, some vaccine droplets were surrounded by intensely labeled macrophages, while the rest were lightly or not stained at all (Figure 4E). Conversely, in animals vaccinated with Silirum^®^, the majority of macrophages surrounding the vaccine droplets showed a more uniform and lighter staining pattern (Figure 4F).

TNF positively immunolabelled cells showed a morphology consistent with macrophages and a cytoplasmic staining pattern, with differences between groups. In the granulomas induced by Gudair^®^, the positive cells were present surrounding only some of the vaccine droplets, but generally, they were located at the periphery of the granulomas (Figure 4G). However, in the granulomas induced by Silirum^®^, the immunolabeled cells were identified in great numbers throughout the inflammatory tissue and surrounding most vaccine droplets (Figure 4H). In both groups, iNOS immunostaining intensity was variable between cells so that the epithelioid macrophages that formed most of the granulomatous infiltrate were weakly stained, but some of those surrounding the necrotic areas, or in the proximity of neutrophils, showed a strong labeling intensity (Figures 4I, J), with no differences between groups.

**Figure 5:**
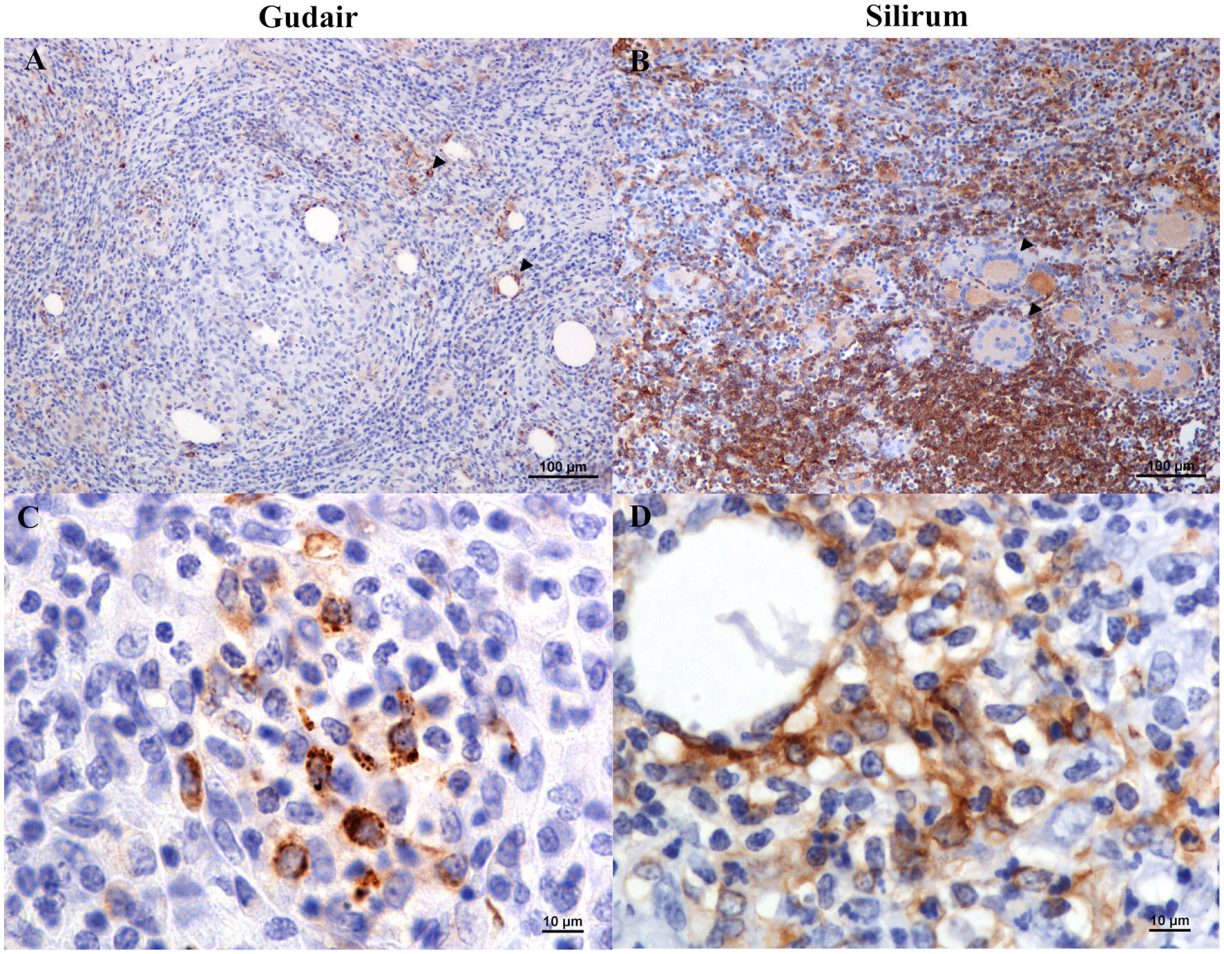
Vaccine-induced granulomas major histocompatibility complex class II (MHC-II) immunolabelling. **(A)** Gudair^®^ vaccine. Some macrophages within the granulomas express MHC-II (arrowheads). However, most epithelioid cells were negative. **(B)** Silirum^®^ vaccine. Positively immunolabelled cells show different intensities of staining: lymphocytes and non-epithelioid macrophages show strong immunolabelling, whereas epithelioid macrophages and Langhans giant cells (arrowheads) show a weaker stain. **(C)** Gudair^®^ vaccine. The staining pattern of MHC-II+ cells was often granular. **(D)** Silirum^®^ vaccine. Strong immunostaining of cells with a dendritic cell morphology surrounding a vaccine droplet.

Positively immunolabelled cells for MHC-II antibody showed a variable pattern. The majority of them were morphologically consistent with macrophages, but some lymphocytes were also stained (Figure 5B), and the staining pattern was often granular (Figure 5C). Epithelioid and Langhans multinucleated giant cells were light to moderately stained (Figure 5B). However, sometimes epithelioid macrophages forming the granulomas were not immunolabelled (Figure 5A), particularly in Gudair^®^ samples. Also, some vaccine droplets were surrounded by intensely labeled cells, which, in some cases, showed a morphology consistent with dendritic cells (Figure 5D), particularly in granulomas induced by Silirum^®^.

**Figure 6:**
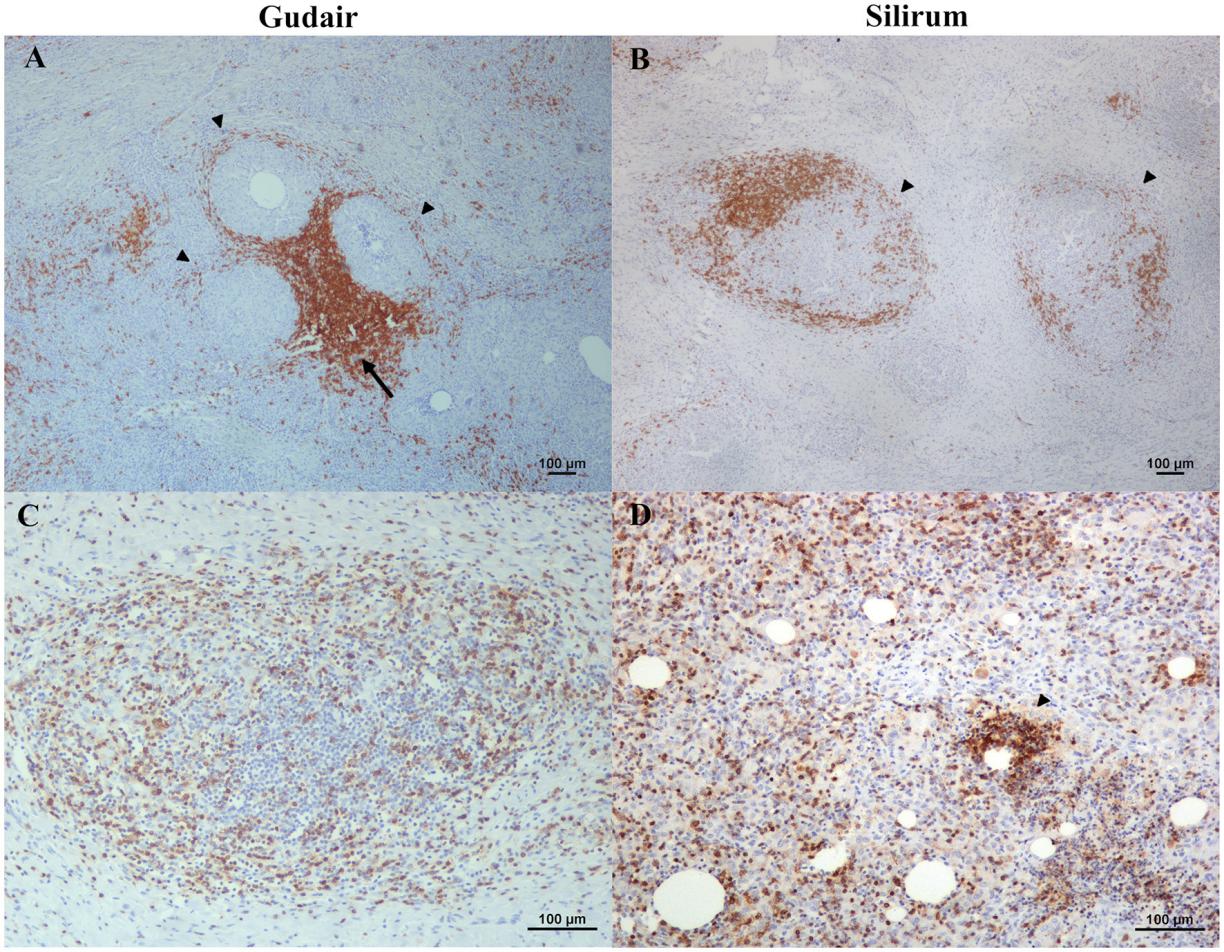
B and T lymphocyte distribution throughout the vaccine-induced granulomas. **(A)** Gudair^®^ and **(B)** Silirum^®^ vaccines. CD20 positively immunolabelled cells form sheaths surrounding the granulomas (arrowheads); sometimes, these sheaths conformed aggregates (arrow) in the confluence of several granulomas. **(C)** Gudair^®^ vaccine. CD3+ positively immunostained cells are scattered uniformly throughout the granulomas, **(D)** Silirum^®^ vaccine. CD3+ cells accumulate around some of the vaccine droplets (arrowhead).

**Figure 7:**
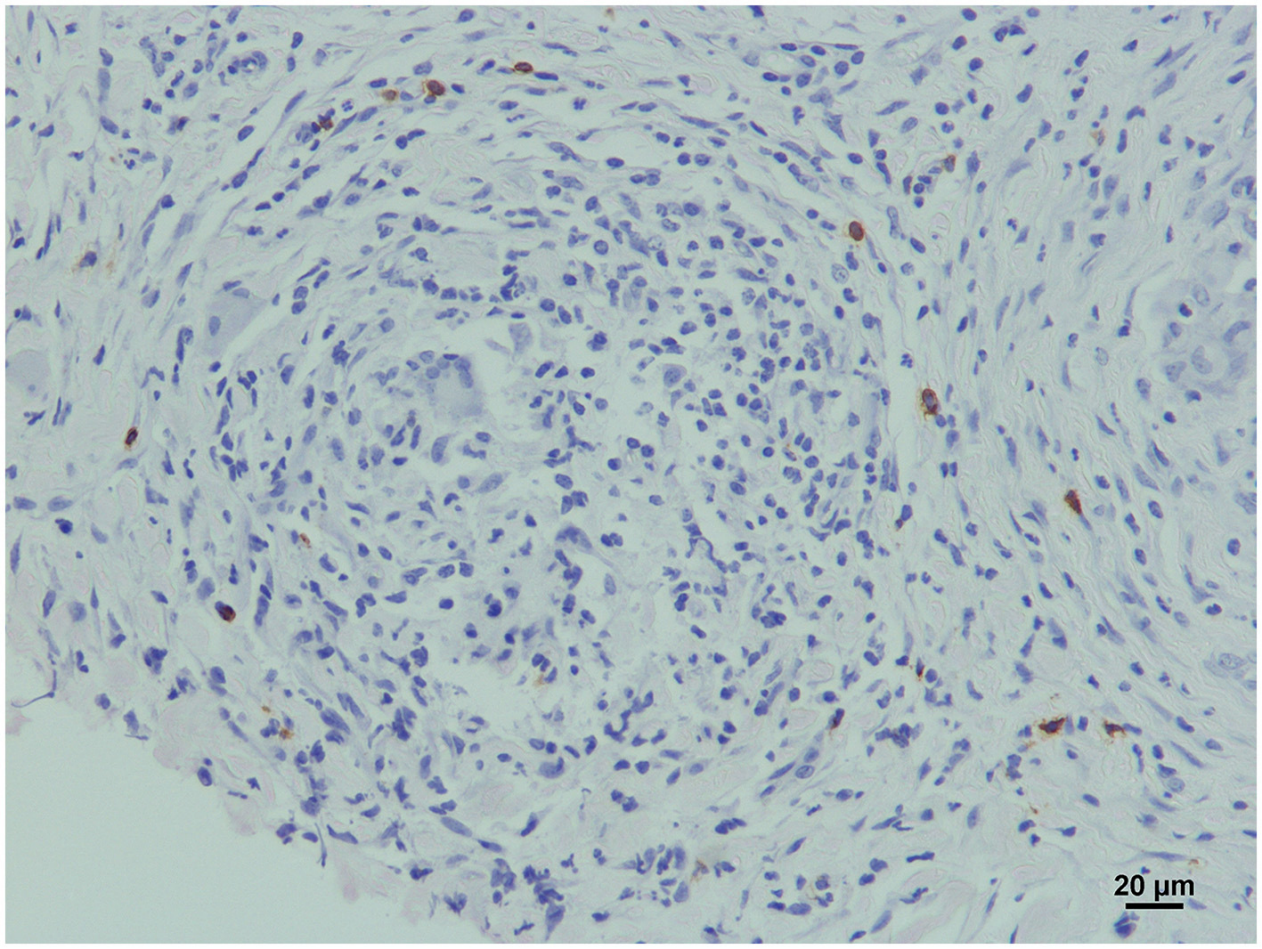
γδ T cells in a vaccine-induced granuloma. WC1 positively immunolabelled cells surround a granuloma induced by Silirum^®^.

Lymphocyte distribution was very similar in all samples, and both CD3 and CD20 immunolabelling patterns were cytoplasmic. CD20+ cells (B lymphocytes) were uniformly distributed, in low quantities, throughout the inflammatory infiltrate, but they were particularly clustered in a well-demarcated layer of variable thickness surrounding the macrophages (Figures 6A, B). This B cell cuff formed the outermost part of the granuloma, and as a result of the merging of several of these outer layers, large aggregates of positive cells could be seen in the confluence of several granulomas (Figure 6A). Most CD3+ cells (T lymphocytes) were scattered throughout the granulomatous infiltrate (Figure 6C), but occasionally they also accumulated in very variable quantities around vaccine droplets (Figure 6D). WC1+ cells (γδ T cells) were very scarce and did not show a specific pattern of distribution, except for the finding of some positive cells in the periphery of a small granuloma from a Silirum^®^ vaccinated animal (Figure 7).

**Figure 8:**
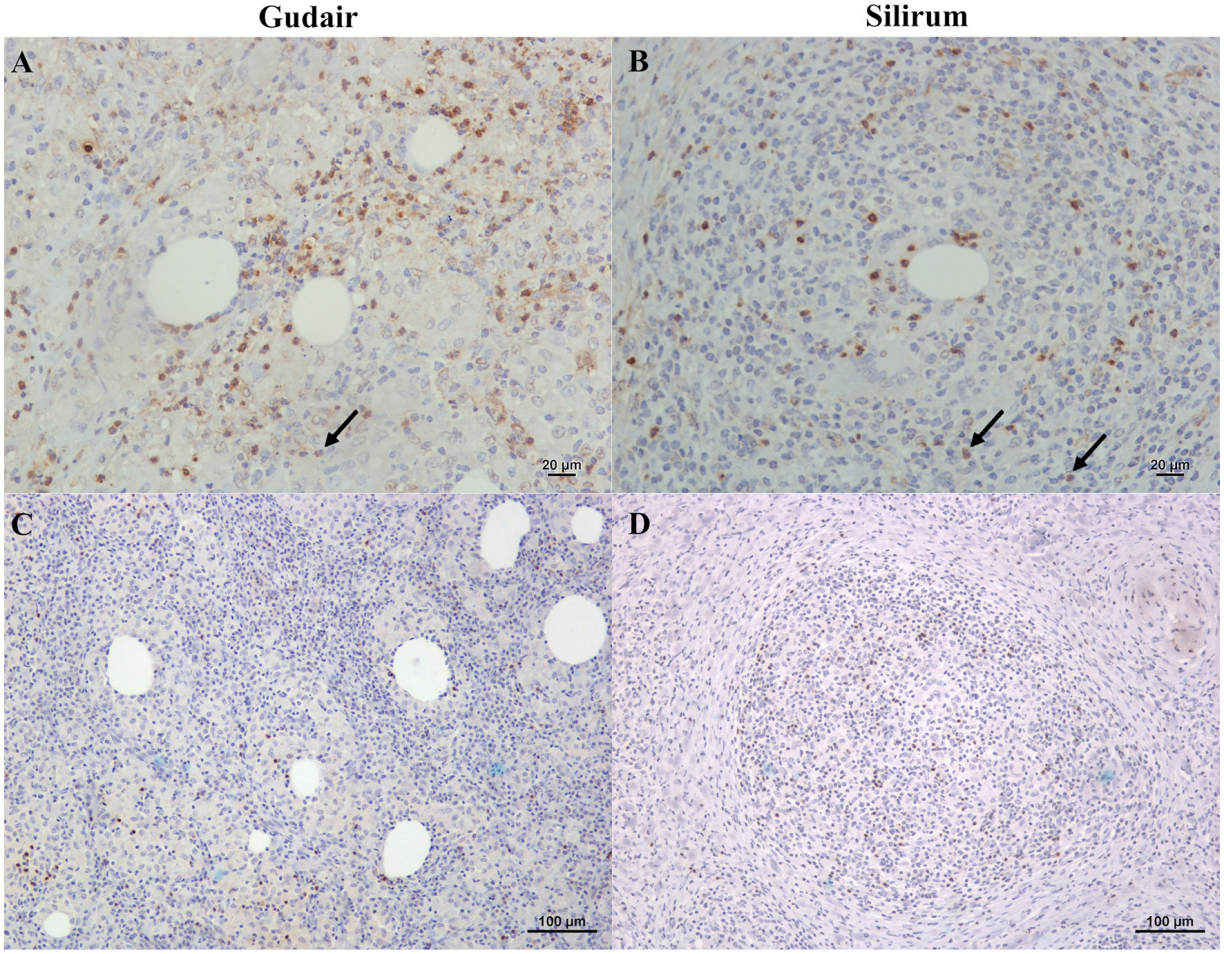
Immunohistochemical expression of IL-4 and IFN-γ within the vaccine-induced granulomas. **(A)** Gudair^®^ and **(B)** Silirum^®^ vaccines. IL-4 positively stained lymphocytes and neutrophils (arrows) were uniformly distributed throughout the granulomatous inflammation in the granulomas induced by both vaccines. **(C)** Gudair^®^ and **(D)** Silirum^®^ vaccines. IFN-γ positively immunolabelled lymphocytes infiltrate the granulomatous tissue in lower numbers.

Lymphocytes and neutrophils expressing IL-4 were uniformly distributed throughout the granulomas and showed a cytoplasmic staining pattern (Figures 8A, B). IFN-γ positively immunostained lymphocytes were present in all samples but in low numbers and did show a similar staining and distribution pattern (Figures 8C, D). Finally, immunohistochemical expression of IL-10 was very low in all groups, with the occasional presence of very few positive lymphocytes.

##### 3.3.2.2 Cell counts

**Figure 9:**
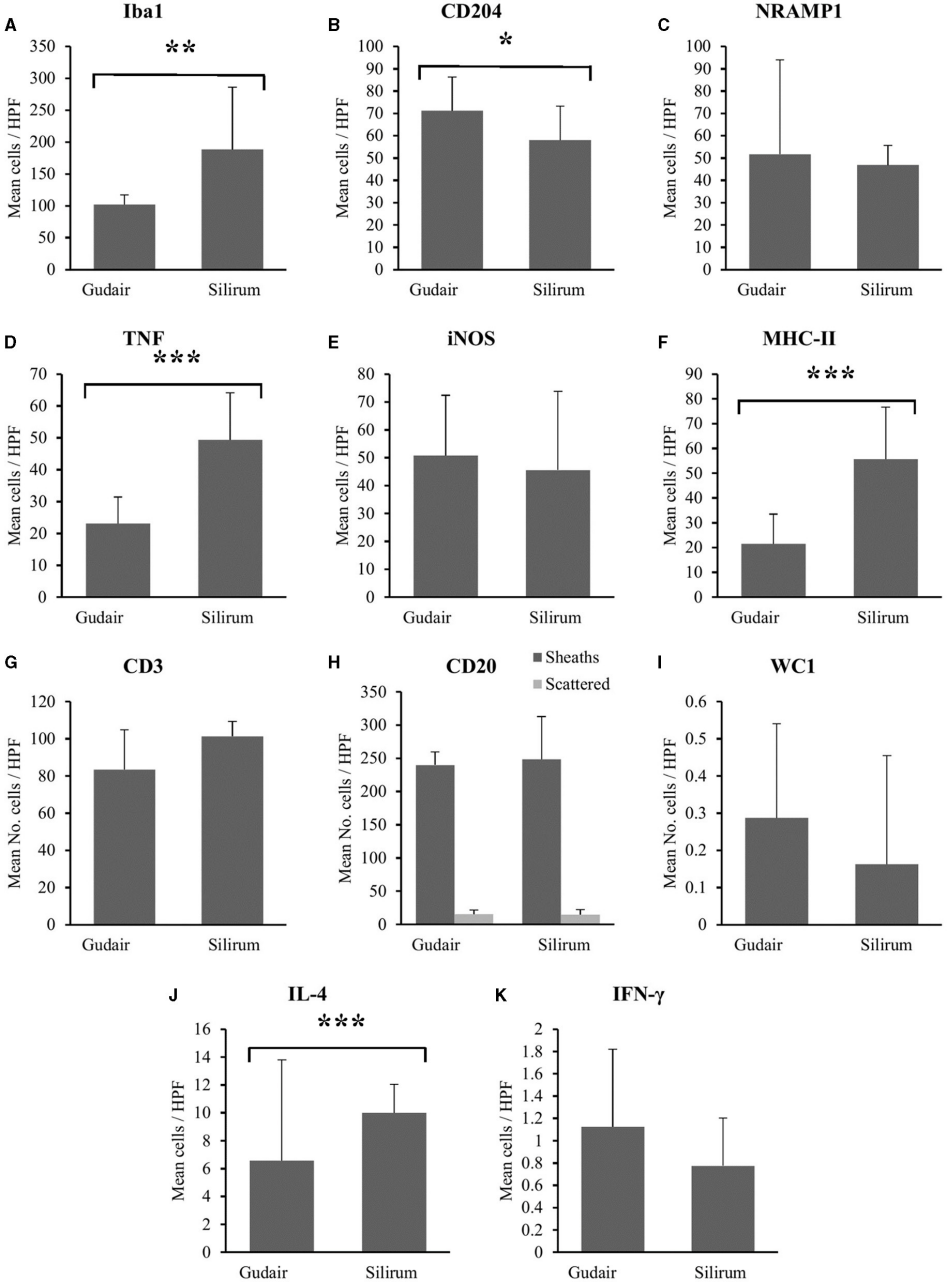
Immunohistochemistry in Gudair^®^ and Silirum^®^ vaccines induced granulomas. The data show the mean number of immunolabeled cells per 400x field for **(A)** Iba1, **(B)** CD204, **(C)** NRAMP1, **(D)** TNF, **(E)** iNOS, **(F)** MHC-II, **(G)** CD3, **(H)** CD20, **(I)** WC1, **(J)** IL-4, and **(K)** IFN-γ. Results are expressed as a mean. Error bars represent the standard deviation of the mean counts between animals. *p < 0.05, **p < 0.01, ***p < 0.001.

In general, the different positively immunolabeled cell subpopulations were identified by the presence, to a greater or lesser extent, of markedly brown staining in their cytoplasm and, sometimes, cell membranes. Variability was observed in the numbers of the different cell populations present in local granulomas according to the vaccine employed. In general, macrophages, variably labeled for the markers Iba1, CD204, NRAMP1, TNF, iNOS, and MHC-II constituted the majority of the cell population present in the inflammatory infiltrate, while T and B lymphocytes were also present, although in smaller numbers. Results of the mean cell counts for each antibody can be seen in Figure 9 and are represented as means and standard deviation. A significant increase in the number of MHC-II (*p* < 0.001), TNF (*p* < 0.001), Iba1 (*p* < 0.01), and IL-4 (*p* < 0.05) positively immunolabelled cells was observed in the animals vaccinated with Silirum^®^ compared to the Gudair^®^ vaccine. A greater number of CD204+, iNOS+, WC1+ γδ T cells, NRAMP1+, and IFN-γ+ cells was observed in the granulomas of animals vaccinated with Gudair^®^ however, this increase was only significant for the marker CD204 (*p* < 0.05). Finally, no significant differences (*p* > 0.05) between groups were found in the number of CD3+ and CD20+ lymphocytes. In the latter, no differences were found either in the number of cells forming a sheath or scattered throughout the granuloma.

## 4 Discussion

In the present study, we performed an extensive histopathological and immunohistochemical study to characterize the inflammatory cell populations present at the injection site granulomas induced by two different commercial vaccines against PTB—Gudair^®^ and Silirum^®^. Additionally, we gathered information on the peripheral immune response developed during the first 75 dpv. It is well-known that vaccination against PTB triggers specific humoral and cellular immune responses (4, 40). It is also known that the intensity and type of the immune response induced by the same antigen can be influenced by the adjuvant employed (16, 41, 42). Consequently, in the absence of other variability factors like environmental influence, age, the antigen used, or previous sensitization to other mycobacteria, the differences observed in the immune response can essentially be linked to the characteristics of the adjuvant employed, given that both vaccines studied share the same *Map* strain and amount.

In all vaccinated animals, the formation of a subcutaneous nodule, similar to that already described in ovine and bovine species (8, 14, 43, 44), was observed at the injection site. In both vaccinated groups, the nodules reached their maximum size at 21 dpv and were still of considerable size by the end of the experiment, an issue that should be considered when vaccinating against PTB. However, differences in size, shape, surface texture, mobility, and consistency were observed between the two vaccines. Overall, the nodules induced by Silirum^®^ showed a better conformation, smaller size, a smaller necrotic center, and less tendency to form fistulae compared to the nodules induced by Gudair^®^.

It has been reported that the development of a strong humoral response following vaccination is crucial to prevent *Map* infection in sheep (45); therefore, though not as important in the control of the infection as the cell-mediated immune response (46–48), it could be an indicator of vaccination effectiveness. In this sense, regardless of the adjuvant employed, the vaccination induced a detectable humoral immune response, already at 15 dpv in some animals, and the main increase in antibody titer took place between 30 dpv and 75 dpv, similar to results found by Pooley et al. (45) and Arteche-Villasol et al. (49) with animals vaccinated with Gudair^®^ reaching the highest titers.

Even though the humoral immune response constitutes a possible indicator of vaccination effectiveness, it has little protective value against mycobacteria and is not sufficient to control mycobacterial infections (50), with the cell-mediated immune response being the most effective. Therefore, the main objective of vaccination is to induce the activity of certain cell populations, which guarantees bacilli destruction and processing at the infection site, either in a direct or indirect way (through cytokine production). Therefore, in the present study, the peripheral immune responses were assessed through IGRA and the PPDa skin tests. As expected, a progressive increase in IFN-γ production by PPDa-stimulated blood in both vaccinated groups took place through the experiment, as had been previously observed for both vaccines (49, 51). Overall, the results of the two assays were coincident, with the animals vaccinated with Silirum^®^ showing a stronger specific cell-mediated response in both techniques employed. However, it should be noted that even though parenteral inactivated vaccines such as Silirum^®^ can significantly influence mucosal immune responses against *Map* (52), the peripheral responses induced by parenteral vaccines do not perfectly align with the mucosal response (53, 54).

Histologically, at the inoculation site, both vaccines induced granulomatous inflammation. However, in granulomas induced by Silirum^®^, a homogeneous distribution of the antigen in smaller adjuvant droplets could be observed. Therefore, it is tempting to hypothesize that this fact, together with the induction of less tissue damage by Silirum^®^, could be responsible for the development of a more specific local immune response. The increased necrosis induced by Gudair^®^ is probably determined by the cytotoxic effects on the cellular membranes of the short-chain fatty acids that constitute the mineral oil and the enzymatic breakdown of native lipid chains into toxic fatty acids caused by the emulsifiers (48, 55). This effect was perhaps limited in Silirum^®^, as its adjuvant is constituted of organic and more refined and biocompatible mineral oils.

In the animals vaccinated with Silirum^®^, a lower amount of antigen could be observed inside the vaccine droplets at the injection site. This was probably caused by the progressive phagocytosis and processing and destruction of the antigen, as it was generally associated with a higher amount of antigen detected inside the epithelioid and giant cells surrounding vaccine droplets and necrotic areas. An interesting finding is that the immunohistochemical staining for *Map* antigens was remarkably superior to the Ziehl–Neelsen method when revealing the antigen presence, as this method depends on the cell wall integrity, which is affected during the inactivation process and is further altered under exposure to the inflammatory microenvironment. For these reasons, immunohistochemistry could be useful in future studies assessing the local antigen presence and their persistence over long periods of time.

There is scarce information about cells present at the granuloma induced by vaccines against mycobacteria; for instance, very recently, CD4, CD8, δ chain, CD79α, CD68, and MHC-II expressing cells were assessed in subcutaneous granulomas induced by *M. bovis* BCG and recombinant derivatives in goats (18). Regarding PTB, a few studies have used immunohistochemistry to evaluate the immune cells present in the subcutaneous granulomas induced by inactivated vaccines or live *Map* (6, 56). Most of this research was focused on the effect of the antigen (live, inactivated, or modified bacteria) and assessed a small number of cell populations (2–3 markers). Hence, the immune response established at the vaccine injection site, and particularly the effect of the adjuvant, are almost unexplored.

One of the most interesting findings observed in our study has been the significantly higher expression of MHC-II positively immunolabelled cells induced by Silirum^®^ at the vaccination nodule, which could be influenced by the nature of its adjuvant that surrounds antigens with a liposome protecting them from protease degradation and denaturation, preserving its three-dimensional structure (57). Further, the differences could be a result of a varying degree of APC activation since it induces the generation of antigen peptide–MHC-II complexes and markedly increases its expression on the plasma membrane (58). Therefore, the lower expression of MHC-II at the Gudair^®^ injection site could be caused by the irregular distribution of the antigen and the persistence of large adjuvant droplets, which induced excessive tissue damage, polarizing macrophage to an epithelioid phenotype. Epithelioid macrophages could be highly phagocytic but do not play an important role in antigen presentation (35). Besides, in the inflammatory infiltrate of both groups, most epithelioid macrophages had an M2 (CD204+) phenotype. M2 macrophages express lower levels of MHC-II than their M1 counterparts (59) and were more abundant in Gudair^®^ than in Silirum^®^ samples (71.2 vs. 58 mean cells per HPF), even though there were significantly more macrophages (Iba1+) in Silirum^®^ samples (101.925 vs. 188.6 mean cells per HPF). The cytoplasmic granular pattern of MHC-II labeling observed in some macrophages could be attributed to endosomes and the antigen-processing compartments (58). In addition to macrophages, in some fields, in the vicinity of vaccine droplets, branched cells strongly labeled with MHC-II could be seen, mainly in Silirum^®^ samples. They were morphologically consistent with dendritic cells, which are potent APCs that stimulate naïve T-cell proliferation (60). Dendritic cells survey the environment with a set of pattern-recognition receptors (PRR), similar to Toll-like receptors (TLR), and, upon recognition, they maturate and differentiate into phenotypes that can stimulate an adaptive immune response. One of those changes is the dramatic increase in surface MHC-II expression (60, 61). Further investigations on this issue, with a more precise characterization of the role of dendritic cell populations in the granulomas, are needed.

Delving deeper into macrophage markers, the more marked membrane pattern staining seen in the proximity of vaccine droplets when labeling Iba1 could be caused by the increased phagocytosis induced by the antigen presence, as Iba1 is a protein involved in membrane ruffling and phagocytosis (62, 63). No differences were found in NRAMP1+ immunolabelled cell numbers, but the variability seen in the intensity could be caused by the differences in antigen distribution. Macrophages showed weak staining with NRAMP1 marker in both groups. In the granulomas induced by Gudair^®^, the expression was very irregular, with macrophages surrounding some of the vaccine droplets expressing high levels of NRAMP1. Those foci of macrophages could be related to the highly concentrated spots of the *Map* antigen, as NRAMP1 becomes associated with the phagosomes upon antigen phagocytosis (30).

Interestingly, no differences were seen in the expression of iNOS+ macrophages in the granulomas between both groups. This enzyme is strongly expressed in granulomas of different etiologies (64) and is associated with a Th1 response. However, significant differences were found in the expression of TNF, a functional M1 marker, which was expressed in higher levels in Silirum^®^ induced granulomas. TNF production by macrophages is induced by stimulated TLR. In this sense, the increase in expression seen in the Silirum^®^ vaccinated animals may be induced by the suggested improved antigen preservation, distribution, and APC activation. This autocrine TNF participates in macrophage activation and stimulates phagocytosis, while exogenous TNF can also activate macrophages previously primed by IFN-γ (65). An increase in TNF expression at the injection site has been described in response to several adjuvants such as MF59, aluminum hydroxide, trehalose-6, 6′-dibehenate -TDB, TLR ligands, and Complete Freund's Adjuvant. Interestingly, the latter, which contains mycobacteria (heat-killed *M. tuberculosis*), induced the highest expression of this cytokine (17).

There were no significant differences in the number and distribution pattern of B (CD20+) and T (CD3+) lymphocytes between the groups. A previous study found that at 3 weeks post-vaccination with Mycopar^®^, B cells were in moderate numbers, and T cells were scarce, while at 6 months post-vaccination, both were abundant at the periphery of the granulomas (66). Therefore, it seems feasible that lymphocyte recruitment mainly takes place after the first month of granuloma formation, and they persist for a long period thereafter. Interestingly, T cells were not described among the epithelioid macrophages of the granuloma in that study, which could be a vaccine-dependent difference. In our study, unlike B cells, T cells were less abundant as part of the lymphocyte cuff, a typical feature of mature mycobacterial granulomas (20), but showed a similar distribution to that observed in cynomolgus macaques (*Macaca fascicularis*) infected with *M. tuberculosis* (67). In this sense, IFN-γ+ immunolabelled cells were also distributed throughout the areas of granulomatous inflammation, but no differences between groups were observed. These IFN-γ producing cells were probably T cells, which are known to infiltrate granulomatous lesions and produce large amounts of Th1-type cytokines, including IFN-γ, contributing to granuloma formation (64, 68, 69). Additionally, the distribution pattern of γδ T cells observed in one of the samples (Figure 7) was very similar to that previously seen in intestinal focal paratuberculous lesions (70). The small size of the granulomas observed in this type of lesion could indicate, as concluded in that previous study, that their distribution at the periphery of the granulomas could participate in the containment of the granulomas, favoring their latency and preventing their progression.

Regarding the expression of interleukins, neutrophils and lymphocytes are known to express IL-4 in response to both live and inactivated *M. tuberculosis*, but IL-10 is produced only in the presence of viable bacteria (71). This expression is driven by TLR signaling, and therefore, the increased IL-4 expression seen in Silirum^®^ samples could be caused by the improved antigen distribution and preservation mentioned before, whereas the absence of live bacteria could explain the limited IL-10 expression in both groups. Also, the local inflammatory environment induced by vaccination does not promote the expression of a classic anti-inflammatory cytokine-like IL-10, which would impair dendritic cell functions like migration and its capability to induce a Th1-type immune response (72–74).

Previous studies have demonstrated the induction of distinct cellular compositions at the injection site granuloma induced by live *Map* and an inactivated *Map* vaccine (6, 56). Overall, the results obtained in the present study show that vaccine adjuvants can also induce significant changes in the immune response established at the injection site. However, studying the cell composition present at the granulomas induced by the adjuvants or the antigen separately would help to understand the mechanisms and possible synergies behind the differences in the response. In fact, the unspecific effect of adjuvants in the peripheral immune response and pathogenesis of paratuberculosis in ruminants has been probed (49). Studying the dynamic interplay between the immune response established at the injection site and its afferent lymph node and drawing robust correlations between this response site and the peripheral and mucosal responses against *Map* would also be of great value. In the present study, granulomas were sampled only at 75 dpv. It would be of interest to study the kinetics of the response at earlier time points when major events in the establishment of the adaptive immune response take place. It would be equally interesting to study later time points given the chronicity of PTB disease, as these injection site granulomas are known to persist for months or even years after vaccination (7).

In conclusion, no systemic adverse reactions were observed in any of the groups. Silirum^®^ induced the formation of a granulomatous nodule comparable in size to that induced by Gudair^®^ but causing a lesser degree of tissue damage, with antigen distributed in smaller vaccine droplets. Also, both vaccines induced an intense humoral and cellular immune response, but Silirum^®^ induced a significantly higher cellular response than Gudair^®^. Regardless of the vaccine type, the inflammatory infiltrates at the injection site are composed of macrophages and lymphocytes, with no clear predominance of any subtype, suggesting that a simultaneous cellular and humoral immune response, with a pro- and anti-inflammatory component, is established at this point. However, a higher number of MHC-II-expressing cells were present in the granulomas induced by Silirum^®^, which could suggest that the enhanced antigen distribution improves antigen uptake and processing by antigen-presenting cells. As an additional conclusion, we found the immunohistochemical staining against *Map* to be far superior to the Ziehl–Neelsen method when assessing the histological distribution of the antigen in vaccine studies. Finally, we can conclude that Silirum^®^ could constitute a good alternative to Gudair^®^ for a *Map* vaccine, achieving similar or better immune response parameters while slightly reducing the tissue damage at the injection site.

## Data availability statement

The raw data supporting the conclusions of this article will be made available by the authors, without undue reservation.

## Ethics statement

The animal study was approved by the Subcomité de Experimentación y Bienestar Animal (OEBA) del Comité de Ética de la Universidad de León, Campus de Vegazana, Calle Profesor s/n 24071, León, Spain. The study was conducted in accordance with the local legislation and institutional requirements.

## Author contributions

MC: Data curation, Formal analysis, Investigation, Writing—original draft. LR: Investigation, Writing—review & editing. JM: Investigation, Writing—review & editing. DZ: Writing—review & editing, Investigation. DG-E: Writing—review & editing. JE: Data curation, Formal analysis, Writing—review & editing. VP: Conceptualization, Funding acquisition, Project administration, Supervision, Writing—review & editing.
